# Comparative Proteomic Analysis of Proteins Involved in the Tumorigenic Process of Seminal Vesicle Carcinoma in Transgenic Mice

**DOI:** 10.1155/2010/726968

**Published:** 2010-05-12

**Authors:** Wei-Chao Chang, Chuan-Kai Chou, Chih-Chiang Tsou, Sheng-Hsiang Li, Chein-Hung Chen, Yu-Xing Zhuo, Wen-Lian Hsu, Chung-Hsuan Chen

**Affiliations:** ^1^Genomics Research Center, Academia Sinica, Taipei, Taiwan; ^2^National Applied Research Laboratories, National Laboratory Animal Center, Taipei, Taiwan; ^3^Institute of Information Science, Academia Sinica, Taipei, Taiwan; ^4^Department of Medical Research, Mackay Memorial Hospital, Tamshui, Taiwan; ^5^Chemistry Department, National Taiwan University, Taipei, Taiwan; ^6^Institute of Atomic and Molecular Sciences, Academia Sinica, Taipei, Taiwan

## Abstract

We studied the seminal vesicle secretion (SVS) of transgenic mice by using one-dimensional gel electrophoresis combined with LTQ-FT ICR MS analysis to explore protein expression profiles. Using unique peptide numbers as a cut-off criterion, 79 proteins were identified with high confidence in the SVS proteome. Label-free quantitative analysis was performed by using the IDEAL_Q software program. Furthermore, western blot assays were performed to validate the expression of seminal vesicle proteins. Sulfhydryl oxidase 1, glia-derived nexin, SVS1, SVS3, and SVS6 showed overexpression in SVS during cancer development. With high sequence similarity to human semenogelin, SVS2 is the most abundance protein in SVS and is dramatically decreased during the tumorigenic process. Our results indicate that these protein candidates could serve as potential targets for monitoring seminal vesicle carcinoma. Moreover, this information can provide clues for investigating seminal vesicle secretion-containing seminal plasma for related human diseases.

## 1. Introduction

Primary seminal vesicle carcinoma is an extremely rare neoplasm; only a few cases have been reported [1, 2]. Seminal vesicle carcinoma is usually associated with diffuse carcinomas of the bladder, prostate, or upper tracts. Very few studies have been published on seminal vesicle invasion [3], and the tumor biology of seminal vesicle carcinoma is not well understood. The diagnoses of seminal vesicle carcinoma are generally based on a combination of morphologic, immunohistochemical, and radiological examinations. However, it is difficult to make a definitive diagnosis using limited biopsy material. 

The seminal vesicle makes up part of the male accessory sexual glands. After puberty, the glands produce a fluid called seminal vesicle secretion (SVS), which accumulates in the lumen of the seminal vesicles. SVS contains both protein and nonprotein components and composes the majority of seminal plasma. The extirpation of seminal vesicles from adult rodents greatly reduces fertility, indicating that SVS plays an important role in sperm activity and modification [4]. Some SVS proteins have been analyzed to characterize their functions and physiological activities [5–9], but the constituents of the SVS proteome have not been well studied.

Proteomic studies are broadly applied to the diagnostic, prognostic, and therapeutic fields. In terms of disease diagnosis and prognosis, body fluid analysis proves to be more attractive than tissue analysis because it provides several advantages including low invasiveness, minimum cost, and easy sample collection and processing [10]. The linear ion trap-Fourier transform ion cyclotron resonance mass spectrometer (LTQ-FT ICR MS) has been exploited as a tool for identifying and characterizing isolated proteins [11]. Combining LTQ-FT ICR MS with liquid chromatography, large-scale and high-quality proteomic analyses of several body fluids are achievable, including tears, urine, and seminal plasma [12–14]. By virtue of its capacity for exhaustive investigation, proteomic research has aroused high expectations for the discovery of biomarkers of various diseases [15, 16].

In this work, we used the transgenic mouse TAg as an animal model to study seminal vesicle carcinoma. Transgenic mouse TAg (C57BL/6-TgN (TRAMP) 8247Ng) expresses an SV40 large T antigen that is driven by the probasin promoter [17]. In addition to being models for prostate cancer, TAg mice also develop other tumors, including neuroendocrine tumors in the prostate and neoplasms in the seminal vesicles. We analyzed the SVS proteome by using one-dimensional PAGE and LC LTQ-FT ICR MS. Accurate masses of tryptic peptides were measured by FT ICR MS, and tandem mass (MS/MS) experiments were performed in LTQ to acquire adequate data for proteomic identification. The resulting mzXML format data and Mascot search results were applied to perform label-free quantitative analysis. Moreover, we used western blot assays to validate the quantitative results.

## 2. Materials and Methods

### 2.1. Animals and Histopathology

TAg mice were purchased from the Jackson Laboratory (Maine, USA), and wild type C57BL/6JNarl normal control mice were obtained from the National Laboratory Animal Center (Taipei, Taiwan). They were housed in a specifically pathogen-free facility and handled in accordance with the guidelines of *Guide for the Care and Use of Laboratory Animals*. All of the mice were given free access to a standard murine chow diet and reverse osmosis water and were maintained on a 14:10 hours light-dark cycle at 21–23°C. In this study, TAg mice (*n* = 21; *n* = 7 for each subgroup) of various ages (32–40 weeks) and normal control mice (*n* = 6) were euthanized and the seminal vesicles and prostate glands were processed for histopathologic examination.

### 2.2. Sample Collection and Preparation

The seminal vesicles were collected by careful dissection to free them from the adjacent coagulating glands, and SVS was squeezed directly into 5 mL of 8 M urea solution. Small amounts of coagulated pellets were removed through centrifugation at 15,000 g for 10 minutes. The protein concentration of the supernatant was determined using the Bio-Rad Protein Assay (Bio-Rad Laboratories, Calif, USA) by the measurement of A_595_. Each 20 *μ*g of SVS proteins was applied to 13% PAGE, and the gel was subsequently visualized through coomassie blue-staining. Gel lanes were divided into 10 sections and all slices were cut into small gel pieces (<1 mm^3^), followed by in-gel digestion. Additionally, five of the observed major bands were cut for independent assays. Briefly, the procedure of in-gel digestion included the following steps in order: (a) destaining with 50% acetonitrile (ACN) and 25 mM ammonium bicarbonate (ABC); (b) reduction using freshly prepared 10 mM dithiothreitol for 45 minutes at 58°C; (c) alkylation using freshly prepared 55 mM iodoacetamide for 45 min at room temperature in the dark; (d) enzyme digestion with 4 ng/*μ*L sequencing grade trypsin in 25 mM ABC solution at 37°C for 16–18 hours; (e) extraction of tryptic peptides using a 60% ACN/1% trifluoroacetic acid solution. After drying to remove the solvent, the re-dissolved tryptic peptides were subjected to LC LTQ-FT ICR MS analysis.

### 2.3. LC LTQ-FT ICR MS Analysis

MS/MS experiments were performed with an LTQ-FT ICR MS (Thermo Electron, Calif, USA) equipped with a nanoelectrospray ion source (New Objective, Mass, USA), an Agilent 1100 Series binary HPLC pump (Agilent Technologies, Calif, USA) and a Famos autosampler (LC Packings, Calif, USA). Tryptic peptide mixtures were injected at a 10 *μ*L/min flow rate into a self packed precolumn in line with a reverse phase C18 nanocolumn (75 *μ*m I.D. × 200 mm) that used Magic C18AQ resin (particle size, 5 *μ*m; pore size, 200 Å; Michrom Bioresources, Calif, USA). The analytic program was set at a linear gradient from 10% to 50% ACN with a 60 minutes running cycle and a split flow rate of 300 nL/min. The full-scan survey MS experiment (*m*/*z* 320–2,000) was executed in FT ICR MS with a mass resolution of 100,000 at *m*/*z* 400. The top ten most abundant multiply charged ions, if they were above a minimum threshold of 1,000 counts, were sequentially isolated for MS/MS by LTQ. Singly charged ions were rejected for MS/MS sequencing.

### 2.4. Mascot Search

The raw files of spectra were converted to mgf files with Mascot Daemon (data import filter: mass range, 600–5400; grouping tolerance, 1.4) and merged into a single file for searching by the MASCOT (version 2.1, Matrix Science Ltd., London, UK) software platform based on the IPI mouse database (v 3.36). The following MASCOT parameter settings were used; the peptide tolerance was 15 ppm with 2^+^ and 3^+^ peptide charges and the MS/MS tolerance was 0.6 Da. Two missed cleavages by trypsin were allowed, carbamidomethyl (C) was used as a fixed modification and oxidation (M) and deamidated (NQ) were used as variable modifications. The significance threshold for the identification was set to *P* < .01.

### 2.5. Label-Free Quantitative Analysis

A software program IDEAL-Q (ID-based Elution time Alignment by Linear regression Quantification) was developed in-house to analyze LC-MS/MS data for label-free quantitative analysis [18]. The program was used to process the LC-MS data and the search results obtained from the Mascot search engine to extract the quantification information. The whole quantitative analysis consisted of the following tasks. (I) Data preparation and construction of the protein list. The raw data files generated from the mass instrument were converted into the mzXML data format by the ReAdW program (http://tools.proteomecenter.org/wiki/index.php?title=Software:ReAdW). The data of each fractional LC-MS/MS runs coming from the same sample were merged and then searched by the Mascot search engine to establish a protein list, which contained identified proteins and their related peptide information. The mzXML files coupled with the peptide and protein identification results were input to the IDEAL-Q program. (II) Extracting quantitative information from each LC-MS run. For quantitative analysis of a peptide in an LC-MS run, we extracted the LC-MS data within the range of ±1.5 minutes of its elution time and ±3.5 Da of the precursor *m*/*z* value. The peak clusters located within the selected elution time and the precursor *m*/*z* value from the extracted data underwent a peptide validation process. For peptide validation, the following three criteria were applied: signal-to-noise ratio (S/N), charge state (CS), and isotope pattern (IP). The S/N criterion checks whether the precursor peak has a valid S/N ratio (>2). The CS criterion eliminated the peak clusters with an incorrect charge state by examining whether the distance between adjacent peaks is equal to 1/*z* (tolerance ±1/10 1/z). Finally, the IP criterion examined the correlation between isotopic distribution of the observed peak intensities and the theoretical isotopic distribution of the peptide. The correlation was then evaluated by a Chi-square goodness of fit test (<0.218). The purpose of peptide validation was to filter out false peptide signals; only the peptide passing the validation criteria were processed to subsequent quantification. We used the extracted ion chromatogram (XIC) to determine peptide abundance in an LC-MS run. (III) Peptide abundance and peptide ratio processing. First we determined the abundances of valid peptides in each LC-MS run. Then we calculated the peptide abundance in a fraction by averaging the peptide abundances of all repeated runs. We summed the peptide abundances in all fractions to represent the peptide quantity in the sample. Following, the peptide ratio between samples could be calculated. (IV) Protein abundance processing. We selected nondegenerate unique peptides and performed Dixon's test to eliminate outliers of peptide ratios for each protein. We then used the top three highly abundant peptides of one protein to represent the quantity of this protein by a weighted average.

### 2.6. Western Blot

Polyclonal antibodies against SVS1, SVS2, SVS3 [19], SVS5, SVS7 [20], sulfhydryl oxidase 1, glia-derived nexin, carcinoembryonic antigen-related cell adhesion molecule 10 (CEACAM 10) [7], Lysozyme C-type M, secreted seminal-vesicle Ly-6 protein 1 (SSLP-1) [21], serine protease inhibitor kazal-like protein (SPINKL) [22], and serine protease inhibitor Kazal-type 3 (SPINK3) [9] were raised in New Zealand White rabbits. Antibody against human albumin, which also crossreacted with mouse albumin, was purchased from Calbiochem (Darmstadt, Germany). After PAGE separation, proteins were transferred to a PVDF membrane using a semidry blotter (ATTA, Tokyo, Japan). The electrophoresis program was set with a constant current (1.5 mA/cm^2^) for 1 hour. The membrane was blocked with 5% (w/v) skim milk in phosphate-buffered saline (PBS) at room temperature for O/N reaction, and then incubated with primary antibody (1 : 5000) in PBS with 2% (w/v) skim milk for 1 hour. After gentle agitation in four changes of PBST (PBS with 0.05% Tween 20) for 15 minutes each, the membrane was immuno-reacted with secondary antiserum (horseradish peroxidase-conjugated goat antirabbit IgG, Amersham Pharmacia) diluted to 1 : 10000 in PBS with 2% (w/v) skim milk for 1 hour. Immuno-reactive bands were revealed using an enhanced ECL substrate according to the manufacturer's instructions (Pierce, Illinois, USA).

## 3. Results

### 3.1. Histopathology and Tumor Categories

**Figure 1:**
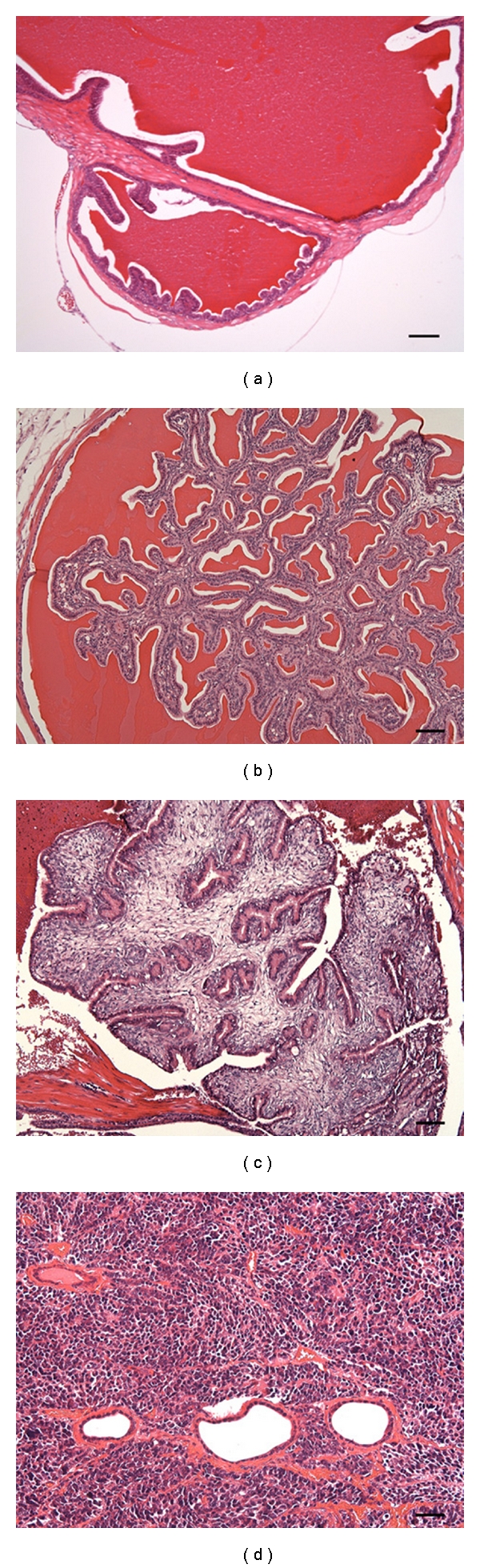
Histopathologic section and tumor categories. Tissue slices from seminal vesicles of normal and various tumor stage mice were stained with hematoxylin-eosin. (a) Normal seminal vesicle, (b) Hp stage seminal vesicle, (c) Ad stage seminal vesicle, and (d) Ac stage prostate with carcinoma. Photographs display the images with original magnification ×10. Scale bar, 75 *μ*m.

According to the histopathologic results, the neoplasm development of the accessory sex gland was categorized into three stages: (I) hyperplasia of the seminal vesicle (Hp), (II) adenoma of the seminal vesicle (Ad), and (III) adenocarcinoma of the seminal vesicle with prostate cancer (Ac) (Figure 1). The Hp stage showed a typical hyperplasia area. These areas, which were characterized by small size and uniformity of cells and nuclei, appeared to have increased glandular enfolding and lack of mitoses. The Ad stage showed the replacement of most of the normal glandular parenchyma with tumor masses encompassing two main components, epithelial cells and stromal cells. The cuboidal to columnar epithelial cells were arranged in multiple papillary fronds and glandular structures, which were separated and supported by abundant, immature, and fibrovascular stroma. However, mitotic figures were infrequent. At the Ac stage, the seminal vesicular tumor showed an appearance similar to the Ad stage, except that sometimes hemorrhage occurred in the seminal vesicles. Prostatic adenocarcinoma of the Ac stage appeared as intraluminar cribriform to papillary epithelial proliferations that completely or almost completely filled the lumen of several adjacent alveoli. The epithelium that lined the tumors was composed of bland-appearing, cuboidal to tall columnar epithelial cells with maintenance of nuclear polarity and sometimes with basophilic cytoplasm.

### 3.2. Protein Pattern and Proteomic Analysis

**Figure 2:**
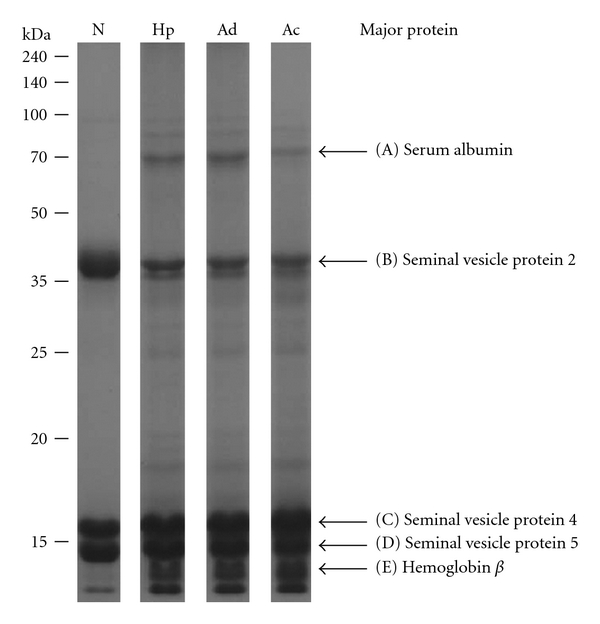
Protein pattern of SVS. SVS proteins were separated by 13% PAGE and visualized by coomassie blue staining. N, normal; Hp, Ad and Ac, various tumor stages. Molecular weights of protein markers are shown on the left. Designated bands A–E were identified by MS/MS, and the names of the major proteins are shown on the right.

Three SVS samples of each group were pooled for the proteomic measurements. SVS proteins were separated by PAGE and their profile was presented by coomassie blue staining (Figure 2). The protein profile was dominated by a small number of highly expressed proteins. The protein distribution of normal SVS was different from those coming from the tumorous SVS, which displayed more complex protein contents. Based on protein pattern comparison, five major bands were chosen as reference indicators. Bands A and E showed upregulated expression, while band B showed downregulated expression in the tumorous SVS. MS/MS was used to analyze the major proteins in bands A to E, which were identified as albumin, seminal vesicle secretory protein 2 (SVS2), SVS4, SVS5, and hemoglobin beta, respectively. The serum proteins, albumin, and hemoglobin beta, are obviously increased in SVS in the early stages of tumorigenesis.

**Figure 3:**
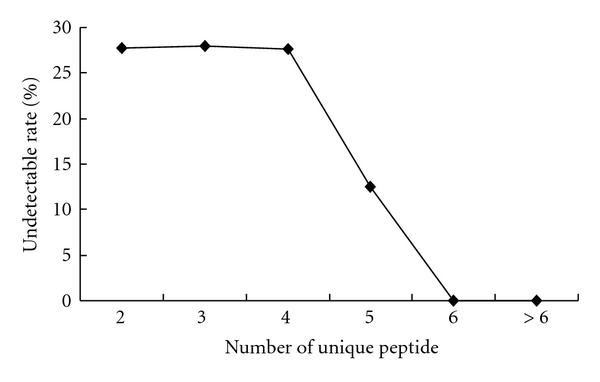
The relationship of unique peptide number and the undetectable rate. The undetectable rate was defined as the percentage of proteins without identifiable peak-areas in the same unique peptide number group.

The proteomic analyses of four groups of SVS, which were normal, Hp, Ad, and Ac, were performed by repeated experimental runs. As a consequence of these analyses, excluding single peptide or single unique peptide matched proteins and keratins that were frequent contaminants in proteomic analysis, 179 proteins met the search criterion and were identified in the proteome by combining four kinds of samples. In order to further improve confidence in the identified proteins, the reproducibility of the matched unique peptides found in every SVS sample was considered as an index to sift through the protein list. The determination of unique characteristics for a peptide was based on the proteotypic peptide sequence that could only match to one protein through the IPI mouse database. The number of unique peptides for one protein was summed with the results of proteomic analyses of the four groups of SVS; therefore, not all peptide signals could be found in the four groups of SVS. Generally, a smaller number of unique peptides coupled with a lower detection rate. We defined the undetectable rate as the average percentage that could not find the matched unique peptides at the designated unique peptide number in the four groups of SVS. With the unique peptide numbers 2, 3, 4, and 5, the undetectable rate was 27.7%, 28.0%, 27.6%, and 12.5%, respectively. When the unique peptide number was more than 5, the undetectable rate decreased to zero (Figure 3). Thus, we chose the unique peptide number 5 as a cut-off criterion to finalize the SVS proteome. Based on this condition, 79 proteins were included in the SVS proteome (Table 1). Among this proteome, 47 proteins were secreted or serum proteins that were the most abundant in this proteome and 16 proteins belonged to seminal vesicle proteins that have been identified or characterized at the previous studies. In addition, a small number of intracellular proteins, including cytoplasm protein, lysosome protein, endoplasmic reticulum protein, and membrane protein were also found in this proteome.

### 3.3. Label-Free Quantitative Analysis

To assess the relative protein expression levels at normal and various cancer stages, quantitative proteomic analysis was pursued by using a label-free quantitative approach, which adopted the average peak-area of the three most intense peptides for the identified proteins to represent the absolute protein abundance [23]. The in-house developed program IDEAL-Q [18] was used to analyze LC-MS data and the corresponding MASCOT search results to extract the quantification information. The results obtained from this quantitative calculation are illustrated in Table 1. The stoichiometric distribution of protein abundance was from 3.26 × 10^4^ (Dickkopf-like protein 1) to 2.77 × 10^9^ (SVS2), which meant that the dynamic range for protein detection reached five orders of magnitude by using the described method. To estimate the applied quantity of SVS proteins for proteomic analysis, the total protein abundances were summed and were 3.31 × 10^9^, 9.74 × 10^8^, 1.05 × 10^9^, and 9.85 × 10^8^ for normal, Hp, Ad, and Ac, respectively. The results showed that the quantity of SVS proteins between various cancer stages was within a 10% difference, but the quantity of normal SVS was three times more than other samples, which appeared to conflict with the gel patterns (Figure 2). We further compared the difference of expression levels of SVS2 and SVS4 (or SVS5) of normal SVS, which displayed almost equal intensity on PAGE gel by dye staining. SVS2 showed a protein abundance of 2.77 × 10^9^, which was about 200-fold higher than SVS4 at 1.29 × 10^7^ (or 150-fold higher than SVS5 at 1.76 × 10^7^). This result implied that quantification errors could occur with the comparison of various molecular weight proteins. To acquire the protein content distribution for realizing the effect of using this quantitative analysis method, the abundance of every protein was converted to the percentage of content in every SVS sample. The results illustrated that SVS2, SVS4, and SVS5 were 83.7%, 0.4% and 0.5% in normal SVS proteins, respectively (Table 1). In the SVS of the Hp stage, the low molecular weight proteins SVS4, SVS5, and hemoglobin beta were 1.4%, 1.2%, and 2.0%, respectively, which were significantly less than SVS2 at 33.0%. Compared with the gel pattern, the levels of low molecular weight proteins tended to be underestimated, which lead to errors in measuring protein quantity or determining the percentage of protein content.

The relative expression levels of tumorous versus normal conditions were achieved by calculating the ratios of absolute SVS protein abundances (Table 1). Among seminal vesicle proteins, SVS1, SVS3, and glia-derived nexin showed upregulated expression with ratios greater than 2. With respect to downregulated expression, lysozyme C and seminal vesicle antigen were decreased two times lower than their normal value. SVS2, with a 10-fold decrease, was the most differentially expressed protein. Similar differences were also observed in the gel patterns. In addition, most serum proteins (35/46, 76%) had an upregulated expression in the tumorous SVS. This phenomenon could relate to the physiological change of seminal vesicles that increases the vascular permeability for serum proteins to enter the lumen of the seminal vesicle.

To evaluate the reproducibility of MS analysis, coefficient of variation (CV) values (%) of the top three highly abundant peptides in repeated runs were calculated and the average CV values are listed in Table 1. In this work, the total average CV values for normal, Hp, Ad, and Ac samples were 16.4%, 15.1%, 10.8%, and 11.5%, respectively.

### 3.4. Western Blot Validation

**Figure 4:**
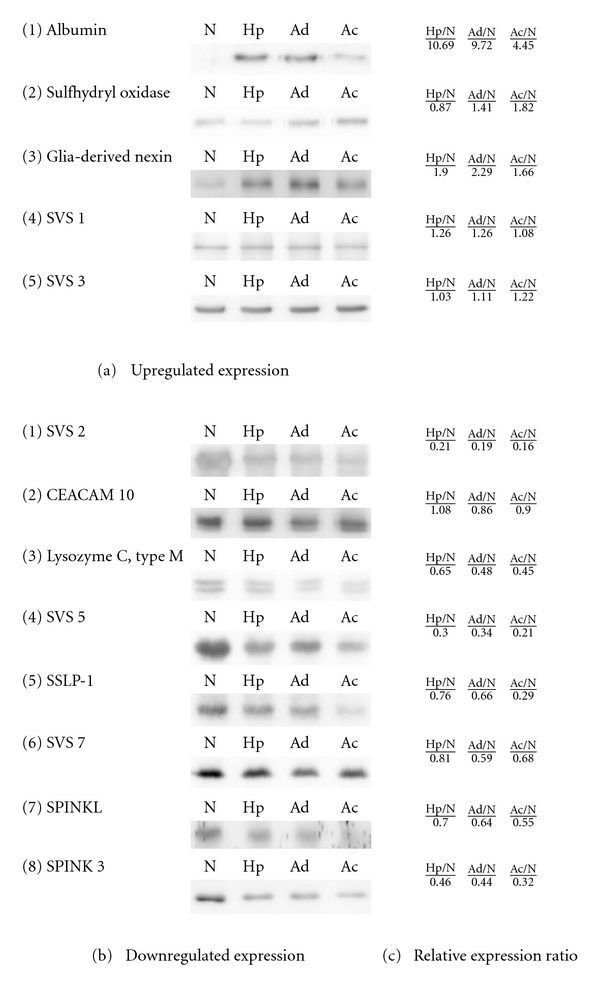
Western blot analysis. The expression of albumin, sulfhydryl oxidase 1, glia-derived nexin, SVS1, SVS3, SVS2, CEACAM 10, Lysozyme C-type M, SVS5, SSLP-1, SVS7, SPINKL, and SPINK3 were examined using polyclonal antibodies. (a) proteins with upregulated expression; (b) proteins with downregulated expression; (c) relative expression ratios compared to normal SVS.

The effects of tumorigenesis on the expression of seminal vesicle secretory proteins were confirmed by western blot analyses, which were also performed to validate the quantification results of the MS analyses (Table 1). Because no protein has been demonstrated to have constitutive or consistent expression in SVS, we used the same loading quantity as the quantitative control in this study. Based on MS quantification, more than 75% of serum proteins showed upregulated expression in the tumorous SVS. Using western blot analysis, for example, the higher levels of albumin were clearly demonstrated in the tumorous SVS, which was consistent with the MS results (Figure 4). Many SVS proteins were also analyzed by the produced antibodies to verify their expression. The signal intensities of western blot were quantitatively measured by the software ImageJ (http://rsb.info.nih.gov/ij/) [24], and the relative expression ratios compared with the normal samples were calculated (Figure 4(c)). Sulfhydryl oxidase 1, glia-derived nexin, SVS1 and SVS3 showed upregulated expression, but SVS2, CEACAM 10, lysozyme C-type M, SVS5, SSLP-1, SVS7, SPINKL, and SPINK3 showed downregulated expression. Regardless of upregulation or downregulation, most SVS proteins changed their secretion depending on the carcinogenesis progress. The protein expression levels revealed a few discrepancies in comparison with the western blot and MS quantification methods. For example, SSLP-1 showed little change during tumorigenic process by using MS analysis; however, it had a clear decreasing pattern by western blot. On the whole, the western blot results were in good agreement with the MS quantification.

## 4. Discussion

### 4.1. Tumorigenesis Affects the Composition of the SVS Proteome

SVS, one of the major contributors to seminal fluid, is important for semen coagulation and plays an important role in promoting sperm motility and suppressing immune activity in the female reproductive tract. Only 16 specific seminal vesicle secretory proteins have been identified with high confidence in the SVS proteome, which is a relatively simple system compared to other body fluids. Almost all SVS proteins make changes in their levels during the tumorigenic process. Compared with increasing protein expression, the suppression effect produced by tumorigenesis on protein expression is more remarkable. The expression of SVS2, SVS5, and lysozyme C-type M decreased 5- to 10-fold at the Ac stage by MS analysis or western blot assay. Although the functions of most SVS proteins in the reproductive system are not fully understood, previous studies have demonstrated that SVS proteins involved in formation of the copulatory plug have the abilities to enhance sperm motility and act as decapacitation factors to modulate the fertilizing ability of spermatozoa. They also have activity of serine protease inhibitors and antimicrobial activity, which may be involved in fertility (Table 2). According to the current study, the physiological functions of SVS proteins did not show a definite correlation with the effects of tumorigenesis. For example, SVS1, SVS2, and SVS3 are the components of copulatory plug formation; however, SVS1 and SVS3 showed upregulated expression, but SVS2 showed downregulated expression. As serine protease inhibitors, glia-derived nexin and SVS6 showed upregulated expression, but SVS5, SPINKL, and SPINK3 showed downregulated expression. Thus, the changes of seminal vesicle proteins during the tumorigenic process need to be explored individually. Based on our studies, almost all seminal vesicle secretory proteins were aberrantly expressed by tumor cells. Only a few seminal vesicle proteins have been demonstrated to correlate with the occurrence of carcinoma (Table 2). The expression of sulfhydryl oxidase 1, glia-derived nexin, and SVS6 increased in seminal vesicle carcinoma and other carcinomas. The results suggest that the expression of these proteins could be under similar regulation in various carcinoma systems.

SVS2, the most abundant protein in SVS, decreased during seminal vesicle carcinoma. SVS2 shows 55% sequence similarity to human semenogelin, which is the major structural component of gelatinous coagulum. Semenogelin is able to protect sperm from protein tyrosine phosphorylation and to prevent induction of the acrosome reaction [38]. A member of the gene family that encodes this semen-coagulating protein is commonly found in mammalian species [6, 39–41]. As a decapacitation factor, SVS2 can bind sperm to affect fertility in the female reproductive tract. Decreased levels of SVS2 at the tumorigenic stages could diminish its inhibition of sperm mobility in a concentration-dependent manner [33]. In addition to varying biological activities, the change of the SVS2 protein level could provide a useful target to monitor the progress of seminal vesicle carcinoma.

### 4.2. Vascular Permeability Contributes to the Elevated Levels of Serum Proteins in SVS

Many serum proteins showed upregulation in the tumorous SVS (Table 1). The most strikingly expressed proteins, albumin and hemoglobin *β*, could be observed on the coomassie blue-stained gel (Figure 2). The expression of albumin was also confirmed by western blot assay (Figure 4). Basal levels of serum proteins could be considered to come from contamination by surgical leaks. However, elevated levels of serum proteins may originate from other sources. First, the mutant secretory cells of the seminal vesicles may produce and secrete them into the lumen, meaning that they are endogenous proteins. Second, angiogenesis or vascular permeability results in the movement of proteins from the blood into the seminal vesicle lumen. To evaluate this phenomenon, we used microarray gene chips to examine the mRNA expression in the seminal vesicles from the four stages. The results showed that the expression of albumin, hemoglobin, apolipoprotein, and *α*1-antitrypsin remained at background levels (data not shown). This suggested that these serum proteins are excluded from being newly synthesized under tumorigenic conditions in seminal vesicles, and that angiogenesis or vascular permeability is responsible for the increase in their levels. Vascular permeability is a tightly regulated process that is often an essential response accompanying angiogenesis, tumor metastasis, or inflammation [42]. Thus, there is a reasonable explanation for late (Ac) stage SVS to have a pink or red appearance. 

### 4.3. Label-Free Quantitative Analysis

Combining one-dimensional PAGE fractionation techniques with LC MS/MS analysis is a popular approach for proteomic research [43, 44]. Due to simplicity and flexibility, the label-free quantitative strategy is an attractive alternative for the quantification of LC MS/MS-based proteomics. In this study, our experimental data were analyzed by using the self-developed program IDEAL-Q, which was designed to reduce the errors in peak detection and drift of retention time, and to validate the selected peptides by the criteria of signal-to-noise ratio, charge state, and isotope pattern. The average CV values were in the range of 10–17% for the top three most abundant peptides, which demonstrated that accurate peak detection and reproducibility could be acquired by using the analytical program IDEAL-Q. However, errors will occur in the analysis of low molecular weight proteins by using the top three most abundant peptides for quantitative analysis, as found in a previous study [38]. The enzymatic reaction produces various peptides with a wide range of ionization efficiency during MS analysis. Smaller proteins will have fewer enzymatic peptides to choose from the highest ionization efficiency region to represent their quantity. Therefore, underestimation is inevitable for the quantification of low molecular weight proteins. For the relative quantification of specific protein abundance changes under various conditions, such kinds of quantification error may not affect the comparison of protein ratios. However, to achieve the accurate absolute quantification, modifying the original results with parameters regarding molecular size needs to be further pursued.

## 5. Conclusion

To obtain SVS samples from humans is quite difficult; therefore, SVS samples from a mouse model system were chosen as an alternative research target, although the volume and composition of SVS differs significantly between various species of mammals. Based on proteomic analysis, we identified 79 proteins, including 16 seminal vesicle secretory proteins, in the SVS proteome. Label-free quantitative analysis was performed to estimate the quantity of the identified proteins. Moreover, most seminal vesicle secretory proteins were subjected western blot assay to further validate their expression. Our data showed that both approaches were in good agreement with each other. We confirmed that many SVS proteins had differential expression profiles during the tumorigenic process, especially SVS2, with a sequence similar to human semenogelin, which was dramatically decreased during the primary stage of seminal vesicle carcinoma. The information obtained from the transgenic mice could be helpful in understanding tumor biology and could be further applied as a reference to study related human diseases.

## Figures and Tables

**Table 1:** Label-free quantitative analysis of mouse SVS proteome.

Accession number			Unique Peptide	(A) protein aboudance				(B) average CV of peptides (%)				(C) relative expression ratio			(D) percentage of content				(E) Western blot assay
	Protein	Mass/pI		**Hp**	**Ad**	**Ac**	**N**	**Hp**	**Ad**	**Ac**	**N**	**Hp/N**	**Ad/N**	**Ac/N**	**Hp**	**Ad**	**Ac**	**N**	
(1) *Seminal vesicle protein*																			

IPI00408931	Carcinoembryonic antigen-related cell adhesion molecule 10	29455/6.84	24	2.43E + 07	2.16E + 07	2.68E + 07	4.29E + 07	12.4	4.3	4.2	10.0	**0.57 **	**0.50 **	**0.63 **	2.50	2.06	2.72	1.30	●
IPI00115065	Glia-derived nexin	44179/9.85	44	4.12E + 06	5.10E + 06	4.68E + 06	2.34E + 06	11.7	7.7	12.5	17.6	**1.76 **	**2.18 **	**2.00 **	0.42	0.49	0.47	0.07	●
IPI00469211	Sulfhydryl oxidase 1, isoform 3	63296/8.17	53	5.00E + 06	7.24E + 06	8.69E + 06	4.98E + 06	7.2	3.6	3.8	14.3	**1.00 **	**1.45 **	**1.74 **	0.51	0.69	0.88	0.15	●
IPI00347009	Secreted seminal- vesicle Ly-6 protein 1	11147/8.72	7	1.49E + 06	2.16E + 06	1.25E + 06	1.35E + 06	31.1	6.3	11.5	22.6	**1.10 **	**1.61 **	**0.93 **	0.15	0.21	0.13	0.04	●
IPI00133448	Seminal vesicle antigen	18266/5.57	26	2.89E + 07	4.27E + 07	2.10E + 07	7.16E + 07	12.0	8.1	5.6	19.7	**0.40 **	**0.60 **	**0.29 **	2.97	4.08	2.13	2.17	
IPI00227548	Seminal vesicle secretory protein 1	93470/8.98	62	9.58E + 06	1.08E + 07	1.40E + 07	3.18E + 06	9.1	4.2	5.7	15.8	**3.02 **	**3.41 **	**4.42 **	0.98	1.03	1.43	0.10	●
IPI00331164	Seminal vesicle secretory protein 2	40824/9.89	203	3.21E + 08	3.25E + 08	2.63E + 08	2.77E + 09	6.7	3.6	4.3	10.9	**0.12 **	**0.12 **	**0.09 **	32.95	31.06	26.67	83.74	●
IPI00227543	Seminal vesicle secretory protein 3 beta	29983/9.37	42	3.32E + 07	3.67E + 07	4.44E + 07	1.64E + 07	9.6	7.9	3.9	13.1	**2.03 **	**2.24 **	**2.71 **	3.41	3.51	4.51	0.49	●
IPI00314341	Seminal vesicle secretory protein 3a	29948/9.39	7	1.16E + 06	1.60E + 06	2.19E + 06	7.24E + 05	28.7	18.1	13.0	12.9	**1.60 **	**2.21 **	**3.03 **	0.12	0.15	0.22	0.02	●
IPI00134286	Seminal vesicle secretory protein 4	12507/8.04	18	1.39E + 07	1.20E + 07	1.73E + 07	1.29E + 07	7.4	7.4	3.9	12.5	**1.08 **	**0.93 **	**1.34 **	1.43	1.15	1.76	0.39	
IPI00135709	Seminal vesicle secretory protein 5	13011/9.52	22	1.20E + 07	1.05E + 07	6.42E + 06	1.76E + 07	8.7	8.5	4.8	14.2	**0.68 **	**0.60 **	**0.36 **	1.23	1.01	0.65	0.53	●
IPI00133431	Seminal vesicle secretory protein 6	11475/5.93	15	1.95E + 06	1.90E + 06	1.48E + 06	1.41E + 06	13.4	8.7	5.8	12.0	**1.38 **	**1.35 **	**1.05 **	0.20	0.18	0.15	0.04	
IPI00877305	Seminal vesicle secretory protein 7	11060/8.93	21	1.03E + 07	1.04E + 07	6.59E + 06	1.47E + 07	6.2	9.5	6.9	14.2	**0.70 **	**0.70 **	**0.45 **	1.06	0.99	0.67	0.45	●
IPI00229083	Serine protease inhibitor kazal-like protein	10535/7.55	6	1.40E + 06	1.39E + 06	8.81E + 05	1.73E + 06	13.2	11.9	12.7	14.5	**0.81 **	**0.81 **	**0.51 **	0.14	0.13	0.09	0.05	●
IPI00132416	Serine protease inhibitor Kazal-type 3	8482/8.26	7	5.58E + 06	7.01E + 06	4.54E + 06	9.53E + 06	4.5	6.3	7.0	7.7	**0.59 **	**0.73 **	**0.48 **	0.57	0.67	0.46	0.29	●
IPI00308892	similar to lysozyme C, type M	17039/9.70	17	4.15E + 06	1.32E + 06	7.05E + 05	5.29E + 06	17.3	32.9	21.1	12.1	**0.78 **	**0.25 **	**0.13 **	0.43	0.13	0.07	0.16	●

(2) *Serum protein and Secreted protein*																			

IPI00131695	Albumin	68648/5.75	109	8.12E + 07	6.21E + 07	3.52E + 07	2.17E + 07	12.4	4.6	4.6	12.1	**3.74 **	**2.86 **	**1.62 **	8.34	5.94	3.57	0.66	●
IPI00129755	Alpha-1-antitrypsin 1-2	45946/5.32	8	7.59E + 05	7.53E + 05	3.16E + 05	1.31E + 06	6.6	6.3	5.5	27.5	**0.58 **	**0.57 **	**0.24 **	0.08	0.07	0.03	0.04	
IPI00123924	Alpha-1-antitrypsin 1-4	45969/5.24	24	8.49E + 06	1.10E + 07	6.45E + 06	7.10E + 06	14.9	6.7	7.1	16.4	**1.20 **	**1.54 **	**0.91 **	0.87	1.05	0.65	0.21	
IPI00123927	Alpha-1-antitrypsin 1-5	45862/5.44	9	8.01E + 05	2.14E + 06	2.17E + 06	3.01E + 06	25.5	4.7	27.1	10.6	**0.27 **	**0.71 **	**0.72 **	0.08	0.20	0.22	0.09	
IPI00117857	Alpha-1-antitrypsin 1-6	45794/5.25	10	1.45E + 06	1.18E + 06	5.62E + 05	3.36E + 05	7.9	7.5	5.8	43.6	**4.32 **	**3.51 **	**1.67 **	0.15	0.11	0.06	0.01	
IPI00128249	Alpha-2-HS-glycoprotein	37302/6.04	9	7.34E + 05	7.96E + 05	7.52E + 05	1.20E + 06	6.0	16.7	9.0	13.9	**0.61 **	**0.66 **	**0.63 **	0.08	0.08	0.08	0.04	
IPI00624663	Alpha-2-macroglobulin	167177/6.38	11	3.69E + 07	4.63E + 07	5.60E + 07	3.66E + 07	15.2	7.1	6.2	15.8	**1.01 **	**1.27 **	**1.53 **	3.79	4.43	5.69	1.11	
IPI00169625	Antiacid phosphatase variable light chain 18	16955/8.50	5	ND	ND	ND	6.34E + 04	ND	ND	ND	0.0	**ND**	**ND**	**ND**	ND	ND	ND	0.00	
IPI00136642	Antithrombin-III	51971/6.10	13	3.27E + 05	5.48E + 05	1.13E + 06	2.61E + 05	32.0	9.2	3.0	12.3	**1.26 **	**2.10 **	**4.35 **	0.03	0.05	0.12	0.01	
IPI00877236	Apolipoprotein A-I	30569/5.64	37	3.99E + 06	5.29E + 06	9.58E + 06	1.05E + 06	15.4	10.2	9.5	20.4	**3.80 **	**5.04 **	**9.13 **	0.41	0.51	0.97	0.03	
IPI00377351	Apolipoprotein A-IV	45001/5.41	23	2.86E + 07	2.82E + 07	3.54E + 07	2.59E + 07	16.7	17.6	19.8	20.7	**1.10 **	**1.09 **	**1.36 **	2.94	2.69	3.59	0.78	
IPI00322463	Beta-2-glycoprotein 1	38593/8.59	9	5.68E + 05	2.74E + 05	3.49E + 05	3.87E + 04	29.5	5.6	8.2	36.4	**14.67 **	**7.08 **	**9.01 **	0.06	0.03	0.04	0.00	
IPI00331286	Beta-2-microglobulin	13770/8.55	15	8.61E + 06	1.19E + 07	9.06E + 06	9.84E + 06	7.5	6.4	6.0	10.0	**0.88 **	**1.21 **	**0.92 **	0.88	1.13	0.92	0.30	
IPI00320420	Clusterin	51623/5.46	47	1.89E + 07	1.06E + 07	2.07E + 07	4.31E + 06	23.8	11.0	10.3	28.0	**4.38 **	**2.45 **	**4.81 **	1.94	1.01	2.11	0.13	
IPI00323624	Complement C3, isoform Long (Fragment)	186538/6.29	53	1.41E + 07	1.62E + 07	1.66E + 07	9.51E + 06	18.4	9.1	9.3	25.8	**1.49 **	**1.70 **	**1.75 **	1.45	1.55	1.69	0.29	
IPI00130010	Complement component factor H	141179/6.85	22	1.17E + 07	1.43E + 07	4.89E + 06	9.29E + 06	14.8	19.1	5.0	15.1	**1.26 **	**1.54 **	**0.53 **	1.20	1.37	0.50	0.28	
IPI00114065	Complement factor B (Fragment)	84951/7.18	9	1.54E + 06	1.56E + 06	1.78E + 06	6.84E + 05	14.4	6.4	4.8	14.2	**2.25 **	**2.27 **	**2.60 **	0.16	0.15	0.18	0.02	
IPI00123744	Cystatin-C	15521/9.18	10	1.03E + 06	1.10E + 06	8.86E + 05	4.57E + 05	8.7	9.9	6.1	33.9	**2.26 **	**2.41 **	**1.94 **	0.11	0.11	0.09	0.01	
IPI00331487	Dickkopf-like protein 1	26623/7.98	6	2.76E + 05	2.86E + 05	1.36E + 05	3.26E + 04	14.3	8.1	23.7	8.7	**8.46 **	**8.77 **	**4.16 **	0.03	0.03	0.01	0.00	
IPI00115522	Fibrinogen, alpha	61288/7.16	13	2.69E + 06	3.24E + 06	6.91E + 06	2.55E + 06	7.2	12.9	9.2	12.3	**1.06 **	**1.27 **	**2.71 **	0.28	0.31	0.70	0.08	
IPI00279079	Fibrinogen, beta	54718/6.68	9	8.79E + 06	7.32E + 06	6.24E + 06	3.98E + 05	22.6	18.5	22.8	13.0	**22.09 **	**18.40 **	**15.67 **	0.90	0.70	0.63	0.01	
IPI00122312	Fibrinogen, gamma	50317/5.38	16	6.52E + 05	5.58E + 05	3.92E + 05	2.45E + 05	14.2	5.0	9.4	6.9	**2.67 **	**2.28 **	**1.60 **	0.07	0.05	0.04	0.01	
IPI00113539	Fibronectin	272319/5.39	11	4.53E + 05	8.32E + 05	5.61E + 05	2.85E + 06	8.0	1.1	12.7	10.6	**0.16 **	**0.29 **	**0.20 **	0.05	0.08	0.06	0.09	
IPI00117167	Gelsolin, isoform 1	85888/5.83	5	1.58E + 06	1.26E + 06	1.40E + 06	4.13E + 05	33.6	22.8	22.4	17.9	**3.84 **	**3.05 **	**3.39 **	0.16	0.12	0.14	0.01	
IPI00124640	Granulin	64972/6.70	17	1.93E + 06	2.19E + 06	2.57E + 06	1.14E + 06	10.5	3.6	15.6	18.6	**1.70 **	**1.92 **	**2.25 **	0.20	0.21	0.26	0.03	
IPI00409148	Haptoglobin	38727/5.88	7	3.24E + 05	3.78E + 05	3.99E + 05	2.44E + 05	2.8	8.7	10.7	13.5	**1.32 **	**1.54 **	**1.63 **	0.03	0.04	0.04	0.01	
IPI00469114	Hemoglobin, alpha	15076/7.96	7	2.99E + 07	3.87E + 07	9.02E + 07	1.39E + 07	7.4	6.9	6.1	24.0	**2.15 **	**2.79 **	**6.49 **	3.07	3.70	9.15	0.42	
IPI00110658	Hemoglobin, beta	15193/8.96	5	1.94E + 07	4.57E + 07	6.68E + 07	1.01E + 07	2.8	15.0	7.9	32.4	**1.92 **	**4.51 **	**6.60 **	2.00	4.36	6.78	0.31	
IPI00128484	Hemopexin	51308/7.92	27	2.18E + 06	8.42E + 06	9.28E + 06	7.59E + 05	19.5	11.5	3.0	7.8	**2.87 **	**11.10 **	**12.23 **	0.22	0.80	0.94	0.02	
IPI00322304	Histidine-rich glycoprotein HRG	60401/7.26	11	1.50E + 07	3.43E + 07	1.65E + 07	4.43E + 07	6.4	4.4	5.2	16.7	**0.34 **	**0.77 **	**0.37 **	1.54	3.28	1.67	1.34	
IPI00114958	Isoform HMW of Kininogen-1	73056/6.05	11	2.56E + 07	2.97E + 07	1.68E + 07	9.15E + 06	14.1	16.4	9.7	19.8	**2.79 **	**3.25 **	**1.83 **	2.63	2.84	1.70	0.28	
IPI00317340	Lactotransferrin	77788/8.86	35	2.63E + 07	2.06E + 07	1.52E + 07	1.81E + 07	26.4	21.3	18.6	18.6	**1.46 **	**1.14 **	**0.84 **	2.70	1.97	1.54	0.55	
IPI00114403	Metalloproteinase inhibitor 1	22613/9.14	5	2.37E + 06	2.26E + 06	2.60E + 06	3.27E + 06	13.4	4.4	6.6	26.0	**0.72 **	**0.69 **	**0.79 **	0.24	0.22	0.26	0.10	
IPI00123223	Murinoglobulin-1	165193/6.00	19	4.28E + 06	6.65E + 06	7.31E + 06	4.64E + 06	5.2	10.5	3.9	18.9	**0.92 **	**1.43 **	**1.57 **	0.44	0.64	0.74	0.14	
IPI00115941	Neutrophil gelatinase-associated lipocalin	22861/8.96	16	2.22E + 06	4.08E + 06	3.67E + 06	1.35E + 06	4.8	5.0	4.8	14.6	**1.65 **	**3.03 **	**2.72 **	0.23	0.39	0.37	0.04	
IPI00309704	Nucleobindin-2	50273/5.05	24	6.56E + 05	2.82E + 06	1.38E + 06	4.88E + 05	9.6	14.1	6.8	16.2	**1.34 **	**5.78 **	**2.83 **	0.07	0.27	0.14	0.01	
IPI00322936	Plasminogen	90723/6.21	16	2.89E + 05	3.11E + 05	2.34E + 05	2.07E + 05	6.2	4.5	4.3	14.3	**1.39 **	**1.50 **	**1.13 **	0.03	0.03	0.02	0.01	
IPI00135156	Protein FAM3B	26135/8.97	11	7.40E + 05	5.28E + 05	5.86E + 05	3.63E + 05	19.9	14.0	7.0	13.8	**2.04 **	**1.46 **	**1.62 **	0.08	0.05	0.06	0.01	
IPI00311104	Semaphorin-3C	85206/8.86	5	3.16E + 05	1.72E + 05	3.22E + 05	6.56E + 04	41.7	27.1	32.4	50.4	**4.82 **	**2.63 **	**4.91 **	0.03	0.02	0.03	0.00	
IPI00131830	Serine protease inhibitor A3K	46850/5.05	19	1.09E + 06	1.12E + 06	5.17E + 05	6.73E + 04	13.5	8.2	4.7	18.0	**16.28 **	**16.63 **	**7.69 **	0.11	0.11	0.05	0.00	
IPI00139788	Serotransferrin	76674/6.94	90	1.46E + 07	1.28E + 07	1.07E + 07	4.87E + 06	13.6	7.6	13.7	16.3	**2.99 **	**2.62 **	**2.20 **	1.50	1.22	1.09	0.15	
IPI00309214	Serum amyloid P-component	26230/5.98	5	5.96E + 05	5.52E + 05	3.30E + 05	5.09E + 05	47.5	36.3	24.6	6.4	**1.17 **	**1.08 **	**0.65 **	0.06	0.05	0.03	0.02	
IPI00321190	Sulfated glycoprotein 1	61381/5.07	8	5.36E + 07	5.04E + 07	5.34E + 07	3.39E + 07	28.0	18.0	5.3	35.8	**1.58 **	**1.49 **	**1.58 **	5.50	4.82	5.42	1.02	
IPI00136556	Transcobalamin-2	47555/5.90	8	9.37E + 05	1.50E + 06	1.85E + 06	1.85E + 06	7.1	4.4	12.7	13.0	**0.51 **	**0.81 **	**1.00 **	0.10	0.14	0.19	0.06	
IPI00127560	Transthyretin	15766/5.77	8	9.05E + 05	1.29E + 06	1.54E + 06	2.16E + 06	12.7	14.9	10.7	9.6	**0.42 **	**0.60 **	**0.71 **	0.09	0.12	0.16	0.07	
IPI00129102	Urokinase-type plasminogen activator	48236/8.53	21	3.09E + 07	2.50E + 07	1.70E + 07	1.74E + 07	11.7	15.7	24.2	20.7	**1.78 **	**1.44 **	**0.97 **	3.18	2.39	1.72	0.53	
IPI00126184	Vitamin D-binding protein	53565/5.39	19	1.22E + 06	1.58E + 06	1.37E + 06	9.77E + 05	16.0	20.5	8.9	14.7	**1.25 **	**1.62 **	**1.40 **	0.13	0.15	0.14	0.03	

(3)* Cytoplasm protein*																			

IPI00110850	Actin, cytoplasmic 1	41710/5.29	11	4.63E + 05	5.79E + 05	3.36E + 05	2.72E + 05	13.0	18.6	5.1	17.1	**1.70 **	**2.13 **	**1.23 **	0.05	0.06	0.03	0.01	
IPI00223757	Aldose reductase	35709/6.71	8	1.21E + 06	1.41E + 06	1.94E + 06	6.78E + 05	20.8	11.6	6.9	8.8	**1.78 **	**2.08 **	**2.86 **	0.12	0.13	0.20	0.02	
IPI00462072	Alpha-enolase	47111/6.37	7	1.81E + 05	1.57E + 05	1.89E + 05	1.44E + 05	47.1	12.2	16.5	6.1	**1.26 **	**1.09 **	**1.31 **	0.02	0.01	0.02	0.00	
IPI00230320	Carbonic anhydrase 1	28303/6.44	5	7.77E + 05	1.14E + 06	2.63E + 05	ND	50.4	1.7	60.3	ND	**ND**	**ND**	**ND**	0.08	0.11	0.03	ND	
IPI00275232	Matrix metalloproteinase 7	30009/6.79	6	9.13E + 05	1.40E + 06	1.36E + 06	2.44E + 05	5.7	12.2	7.2	11.1	**3.74 **	**5.75 **	**5.55 **	0.09	0.13	0.14	0.01	
IPI00121788	Peroxiredoxin-1	22162/8.26	9	1.90E + 05	1.92E + 05	2.86E + 05	1.76E + 05	14.7	8.2	7.0	19.9	**1.08 **	**1.09 **	**1.62 **	0.02	0.02	0.03	0.01	

(4)* Lysosome protein*																			

IPI00111013	Cathepsin D	44925/6.71	6	2.14E + 05	1.69E + 05	3.55E + 05	2.82E + 05	12.7	10.7	15.8	12.7	**0.76 **	**0.60 **	**1.26 **	0.02	0.02	0.04	0.01	
IPI00128154	Cathepsin L1	37523/6.37	7	7.14E + 05	1.80E + 06	1.38E + 06	5.97E + 05	8.6	7.8	15.8	17.7	**1.20 **	**3.02 **	**2.31 **	0.07	0.17	0.14	0.02	
IPI00311305	Deoxyribonuclease-2-beta	40768/9.49	16	6.69E + 05	6.34E + 05	4.55E + 05	9.34E + 05	9.6	11.4	25.8	20.8	**0.72 **	**0.68 **	**0.49 **	0.07	0.06	0.05	0.03	
IPI00381303	Lysosomal alpha-mannosidase	114532/8.30	24	4.99E + 06	4.56E + 06	5.26E + 06	6.23E + 06	11.4	11.2	7.0	19.7	**0.80 **	**0.73 **	**0.84 **	0.51	0.44	0.53	0.19	

(5)* Endoplasmic Reticulum protein*																			

IPI00319992	78 kDa glucose-regulated protein	72377/5.07	13	4.17E + 05	4.91E + 05	2.11E + 05	1.42E + 05	28.5	25.1	29.8	9.8	**2.93 **	**3.45 **	**1.48 **	0.04	0.05	0.02	0.00	
IPI00187353	ERO1-like protein beta	53484/8.39	13	1.35E + 05	1.55E + 05	8.01E + 04	3.52E + 05	11.6	10.4	26.3	8.0	**0.38 **	**0.44 **	**0.23 **	0.01	0.01	0.01	0.01	
IPI00138342	Liver carboxylesterase N	61133/5.10	8	5.41E + 05	8.27E + 05	5.24E + 05	1.83E + 06	6.6	14.4	15.1	12.2	**0.30 **	**0.45 **	**0.29 **	0.06	0.08	0.05	0.06	
IPI00135686	Peptidylprolyl isomerase B	23699/9.56	14	7.22E + 06	9.49E + 06	4.73E + 06	4.16E + 06	10.2	5.9	7.2	13.0	**1.74 **	**2.28 **	**1.14 **	0.74	0.91	0.48	0.13	

(6) *Membrane protein*																			

IPI00553419	Desmoplakin	332706/6.43	10	7.42E + 06	6.91E + 06	5.89E + 06	5.74E + 06	18.3	16.4	9.6	13.2	**1.29 **	**1.20 **	**1.03 **	0.76	0.66	0.60	0.17	
IPI00881007	Endothelial chloride channel	99794/6.72	5	1.87E + 05	2.15E + 05	1.12E + 05	4.45E + 04	16.6	10.1	27.9	12.4	**4.21 **	**4.83 **	**2.52 **	0.02	0.02	0.01	0.00	

**Table 2:** Summary of seminal vesicle secretory proteins identified in mouse SVS.

			Expression		
Protein	Function	Reference	Mass	Western	Cancer-related studies
*Upregulated expression*					

Sulfhydryl oxidase 1	Catalyze disulfide bond formation	[25]	⇑	⇑	[26, 27]
Glia-derived nexin	Serine protease inhibitor	[8]	⇑	⇑	[28, 29]
Seminal vesicle secretory protein 1	Amine oxidase activity/Copulatory plug formation	[5]	⇑	⇑	—
Seminal vesicle secretory protein 3a	Copulatory plug formation	[19]	⇑	⇑	—
Seminal vesicle secretory protein 3*β*	Copulatory plug formation	[30]	⇑	⇑	—
Seminal vesicle secretory protein 6	Serine proteinase inhibitor	[31]	⇑	—	[32]

*Downregulated expression*					

Seminal vesicle secretory protein 2	Semen-coagulating protein/Sperm decapacitation factor	[6, 33]	⇓	⇓	[34]
Carcinoembryonic antigen-related cell adhesion molecule 10	Sperm binding and enhancement of sperm motility	[7]	⇓	⇓	—
Lysozyme C, type M	Primary bacteriolysis	—	⇓	⇓	—
Seminal vesicle secretory protein 5	Serine proteinase inhibitor	[35]	⇓	⇓	—
Secreted seminal-vesicle Ly-6 protein 1	Potential cellular adhesion and signaling	[21]	⇔	⇓	—
Seminal vesicle secretory protein 7	Sperm capacitation factor	[20]	⇓	⇓	—
Serine protease inhibitor kazal-like protein	Serine protease inhibitor/Sperm decapacitation factor	[22]	⇓	⇓	—
Serine protease inhibitor Kazal-type 3	Serine protease inhibitor/Sperm decapacitation factor	[9]	⇓	⇓	—
Seminal vesicle antigen	Sperm decapacitation factor	[36]	⇓	—	—
Seminal vesicle secretory protein 4	Anti-inflammatory and immune-modulating agent	[37]	⇔	—	—
